# Unraveling the Complex Interplay Between Transcription Factors and Signaling Molecules in Thyroid Differentiation and Function, From Embryos to Adults

**DOI:** 10.3389/fendo.2021.654569

**Published:** 2021-04-20

**Authors:** Arístides López-Márquez, Carlos Carrasco-López, Celia Fernández-Méndez, Pilar Santisteban

**Affiliations:** ^1^ Instituto de Investigaciones Biomédicas “Alberto Sols”, Consejo Superior de Investigaciones Científicas (CSIC) y Universidad Autónoma de Madrid (UAM), Madrid, Spain; ^2^ Laboratorio de Investigación Aplicada en Enfermedades Neuromusculares, Unidad de Patología Neuromuscular, Servicio de Neuropediatría, Institut de Recerca Sant Joan de Déu, Esplugues de Llobregat, Spain; ^3^ Centro de Investigación Biomédica en Red (CIBERONC), Instituto de Salud Carlos III, Madrid, Spain

**Keywords:** transcription factors, signaling pathways, development, differentiation, thyroid

## Abstract

Thyroid differentiation of progenitor cells occurs during embryonic development and in the adult thyroid gland, and the molecular bases of these complex and finely regulated processes are becoming ever more clear. In this *Review*, we describe the most recent advances in the study of transcription factors, signaling molecules and regulatory pathways controlling thyroid differentiation and development in the mammalian embryo. We also discuss the maintenance of the adult differentiated phenotype to ensure the biosynthesis of thyroid hormones. We will focus on endoderm-derived thyroid epithelial cells, which are responsible for the formation of the thyroid follicle, the functional unit of the thyroid gland. The use of animal models and pluripotent stem cells has greatly aided in providing clues to the complicated puzzle of thyroid development and function in adults. The so-called thyroid transcription factors – Nkx2-1, Foxe1, Pax8 and Hhex – were the first pieces of the puzzle identified in mice. Other transcription factors, either acting upstream of or directly with the thyroid transcription factors, were subsequently identified to, almost, complete the puzzle. Among them, the transcription factors Glis3, Sox9 and the cofactor of the Hippo pathway Taz, have emerged as important players in thyroid differentiation and development. The involvement of signaling molecules increases the complexity of the puzzle. In this context, the importance of Bmps, Fgfs and Shh signaling at the onset of development, and of TSH, IGF1 and TGFβ both at the end of terminal differentiation in embryos and in the adult thyroid, are well recognized. All of these aspects are covered herein. Thus, readers will be able to visualize the puzzle of thyroid differentiation with most – if not all – of the pieces in place.

## Introduction

Consistent with its important hormonal role in embryonic development, the thyroid gland is one of the first endocrine organs to differentiate. In mammals, the thyroid forms as an evagination of endodermal epithelial cells on the median surface of the developing pharyngeal floor. Two main cell types characterize the thyroid gland: epithelial or follicular cells and the parafollicular cells. The former, also known as thyrocytes, represent the majority of cells and are responsible for the synthesis and secretion of the thyroid hormones. The latter, also termed C-cells, are responsible for calcitonin synthesis and secretion. For many years it was believed that these two thyroid cell types derived from endoderm and neural crest, respectively (1). However, it is currently accepted that both are of endodermal origin (2), with the C-cell precursors arising from the ultimobranchial bodies that derive from the ventral recess of the fourth pharyngeal pouch (3).

In this review, we will focus on the follicular cells in mammals, with some illustrative examples from zebrafish and chicken, as these animal models have contributed substantially to our understanding of thyroid development. Polarized thyroid follicular cells, with a basal and an apical membrane, are responsible for the formation of the thyroid follicles. After its specification and budding in the pharynx floor, the thyroid primordium undergoes a series of events that involve complex morphological and differentiation processes (4). First, the thyroid follicular progenitor cells expand ventrally, as a diverticulum, and begin to migrate caudally downward from the bud to their tracheal position. Once the thyroid reaches its final position on both sides of the trachea, the thyroid lobes enlarge to form the distinctive bilobed (butterfly) shape. Subsequently, folliculogenesis takes place, which is a complex and essential mechanism for the terminal differentiation program, and occurs only when migration is complete and the follicles are formed.

**Figure 1:**
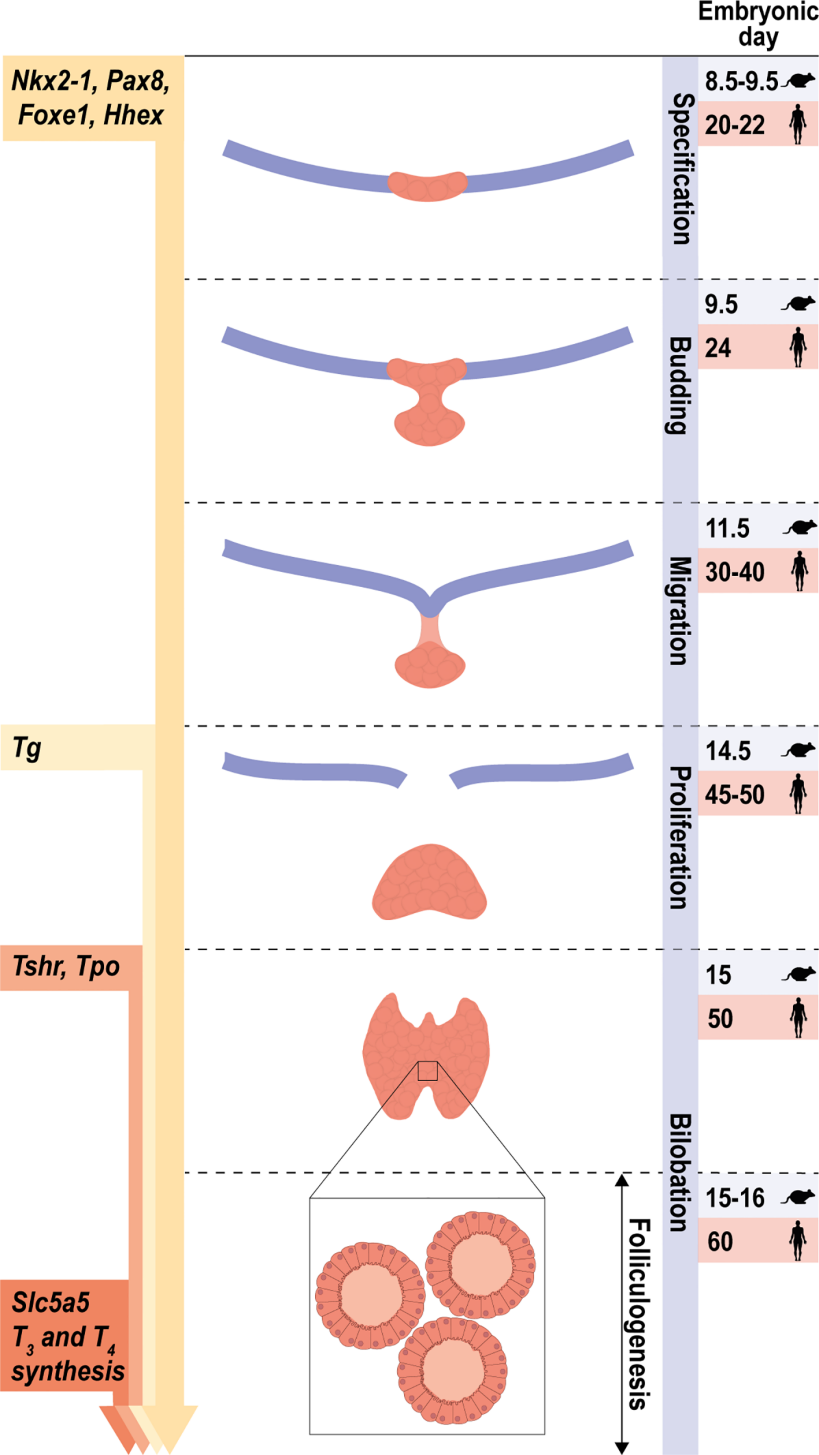
Stages of early thyroid development. Schematic representation of the main steps of thyroid morphogenesis indicating the corresponding embryonic or post-fertilization day in mice and humans, respectively. From an undifferentiated endoderm, the co-expression of *Nkx2-1*, *Foxe1*, *Pax8* and *Hhex* defines the specification of thyroid progenitor cells. Arrows on the left represent the initial stage at which the expression of the thyroid transcription factors and their main target genes are first detected. The onset of thyroid hormone synthesis and *Slc5a5* expression occurs at the final stage, once folliculogenesis has occurred and the thyroid gland is completely functional.

**Figure 2:**
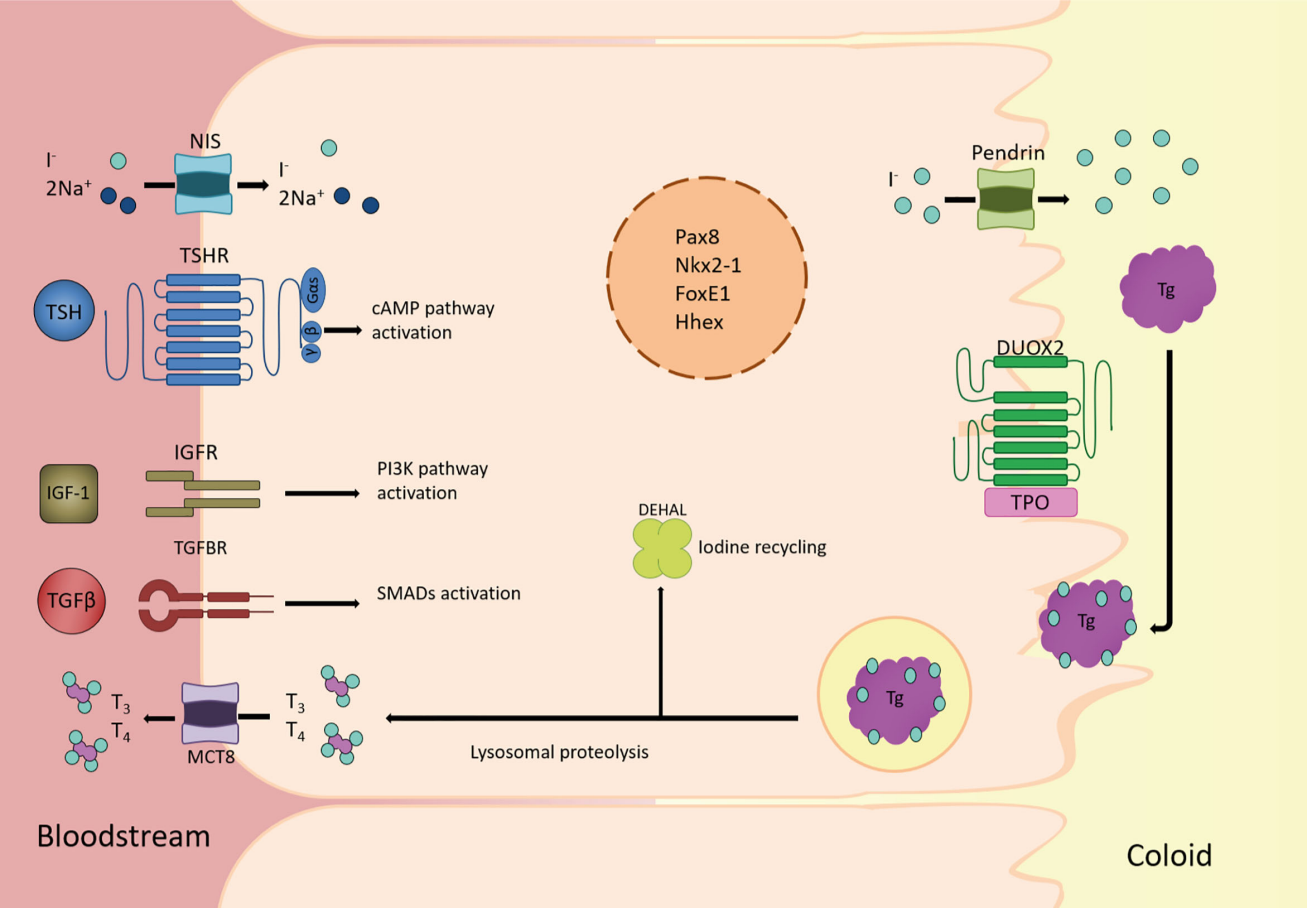
Overview of the main players in thyroid follicular cell function. Schematic diagram of a thyroid follicular cell and the main proteins involved in the synthesis, storage, and release into bloodstream of the thyroid hormones triiodothyronine (T3) and thyroxine (T4). At the basolateral membrane, circulating iodide (I^-^) is actively taken-up by the Nis symporter. It then passively diffuses to the apical membrane where it is actively transported to the lumen by Pendrin and other transporters. Once I^-^ is oxidized by thyroid peroxidase (Tpo), using H_2_O_2_ generated by the oxidase Duox2, tyrosine residues in thyroglobulin (Tg) are iodinated to form mono-iodotyrosine (MIT) and/or di-iodotyrosine (DIT), whose coupling yields thyroid hormones. Colloid droplets rich in Tg are endocytosed into the thyroid follicular cell, where Tg lysosomal proteolysis releases MIT, DIT, T4, and T3. Thyroid hormones are transported to circulation by the monocarboxylate transporter 8 (Mct8), whereas I^-^ from MIT and DIT is recycled by the iodotyrosine deiodinase (Dehal1). All of these processes rely on the co-existence of the thyroid transcription factors Nkx2-1, Foxe1, Pax8 and Hhex, in the nucleus of the thyroid follicular cell, from where they regulate the expression of genes such as *Slc5a5, Tpo, Tg* and *Tshr.* Also, in the basolateral membrane, TSH, IGF1 and TGFβ trigger the main pathways involved in adult maintenance of thyroid differentiation by cAMP, PI3K and SMAD activation, respectively.

At this time, thyroid cells start to express the thyroglobulin (*Tg*), thyroperoxidase (*Tpo*), TSH receptor (*Tshr*) and sodium (Na) iodide (I) symporter NIS (*Slc5a5*) genes, which are considered as the thyroid differentiation genes in this review. Together with other genes including dual oxidase 2 (*Duox2*), the membrane transporters pendrin *(Slc26a4)*, monocarboxylate transporter 8 Mct8 (*Slc16a2*) and dehalogenase Dehal1 (*IYD*), these differentiation genes are required for the biosynthesis of the thyroid hormones triiodothyronine (T3) and tetraiodothyronine/thyroxine (T4). All stages of thyroid development are depicted in **Figure 1**, with the indication of the observed times at which they are expressed in mouse and human; the genes expressed in a differentiated thyroid follicular cell are depicted in **Figure 2**.

Differentiated thyroid epithelial cells form follicular structures surrounding a lumen, with the resulting follicle considered as the functional unit of the thyroid gland. Recent studies, however, have revealed that the elements that comprise the functional unit of the thyroid are more complex than originally thought. In this regard, the concept of an angiofollicular unit has been proposed, which also includes endothelial cells of the blood vessels that sheath the thyroid follicles, and which is formed during embryonic development. The intimate relationship between the blood microvasculature and the thyroid follicle is essential for thyroid homeostasis and function (5). Differentiation is sustained throughout life, and must be finely regulated to maintain a controlled production of thyroid hormones. The mechanisms that control thyroid differentiation in the embryo occur simultaneously, with cross-talk between all the actors playing a role in this finely orchestrated process. To provide a more systematic and didactic review, we will organize the work by first defining the role of the thyroid transcription factors (TTFs) and then the signals that regulate the different steps in thyroid development. We will discuss the most current knowledge of new players in all the processes involved in achieving a differentiated thyroid functional unit in the embryo and the adult.

We will mainly refer to data obtained from mice as the most recent advances come from studies in this model system. However, we will translate this information to humans when possible. We will follow the standard nomenclature; thus, proteins will be referred to in capital letters for humans and a capital followed by lowercase for mice. Italics will be used when referring to genes.

## Thyroid Transcription Factors

**Figure 3:**
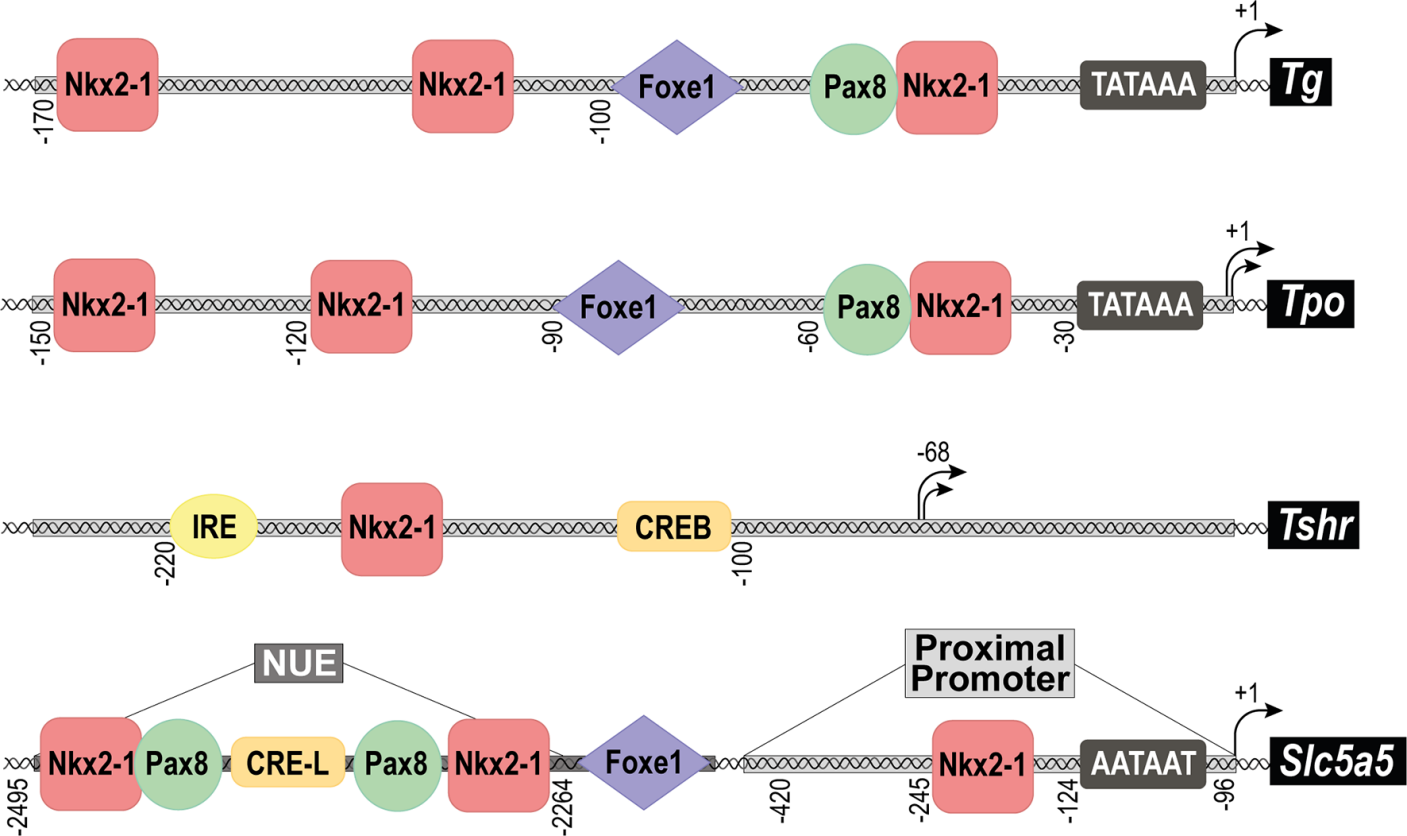
Structure of the promoter regions of the main thyroid differentiation genes. Schematic representation of the main transcription factor binding sites found in the promoter regions of the genes: *Thyroglobulin* (*Tg*), *Thyroid peroxidase* (*Tpo*), *TSH receptor* (*Tshr*) and *Sodium/iodide symporter* (*Nis, Slc5a5*). In the case of *Slc5a5*, the proximal promoter, and the Nis Upstream Enhancer (NUE) are separately indicated. Black arrows represent the transcriptional initiation site of each promoter and numbers define its position. The TATA box is indicated as well as the binding sites for transcription factors: Nkx2-1, Foxe1, Pax8, CREB, CRE-L (CRE-Like) and IRE (Insulin Respose Element).

Differentiated follicular cells express four genes encoding the so-called TTFs: these are *Nkx2-1* (formerly known as thyroid transcription factor 1, *Ttf1* or *T/ebp*), *Foxe1* (formerly known as thyroid transcription factor 2, *Ttf2*), *Pax8* and *Hhex* (hematopoietically-expressed homeobox protein) (4, 6, 7). These genes are members of the forkhead-, paired- and homeo-box family of transcription factors, and were initially identified through their promoter binding sites on thyroid differentiation genes such as *Tg, Tpo, Tshr* or *Slc5a5* (7–9) (**Figure 3**). Most of the knowledge on the TTFs derives from the use of mouse models, embryonic stem (ESCs), induced pluripotent stem (iPSCs) and differentiated cell cultures, and advances in the understanding of their regulation and function have contributed to establishing the molecular mechanisms involved in thyroid differentiation. Although there are many reviews on TTFs, below we will provide a brief summary of each one, focusing mainly on its role in the complex differentiation processes that occur from embryos to adulthood for the correct function of the thyroid gland.

### Nk2 Homeobox 1, Nkx2-1

The homeodomain transcription factor *Nkx2-1* was initially cloned and identified in rat thyroid cells and in mouse thyroid (10, 11), and was later observed to be widely distributed in other organs. It is expressed in both follicular and parafollicular cells, and also in other tissues such as lung, forebrain, and hypothalamus (12). Its expression in the mouse endoderm at embryonic day (E) 8.5 coincides with the recruitment of the cells from the adjacent endoderm that ultimately form the thyroid bud, prior to migration, and it remains expressed along embryonic development and in adult life. *Nkx2-1*-null mice die at birth from impairment of lung organogenesis and lack thyroid and pituitary glands, and exhibit severe abnormalities in the ventral forebrain (13). Although the thyroid is absent in *Nkx2-1*-null mice the bud still forms, but disappears later by apoptosis, pointing to a role for *Nkx2-1* in the survival of thyroid precursor cells but not in their specification (14). In mice in which *Nkx2-1* expression is inhibited at later stages of embryonic development, a structure can be observed with abnormal thyroid follicles, indicating that *Nkx2-1* plays an important role in maintaining tissue architecture and cell function in the adult thyroid (15), as we will discuss later in the follicle formation section.

### Forkhead Box E1, Foxe1

*Foxe1* was initially cloned and identified in rat thyroid cells, and encodes a transcription factor containing a forkhead domain (16). Like *Nkx2-1*, it has a broad expression pattern in other embryonic tissues such as tongue, epiglottis, palate, hair follicles and pituitary, among others. It has also been detected in many adult organs, including testis, brain, pancreas kidney and intestine (7). *Foxe1* is expressed at E8.5 in the endoderm lining the foregut and also in the cells that form the thickening primordium in the midline of the pharyngeal floor in the embryonic mouth cavity. Accordingly, its expression in the embryo is more evident in the posterior pharynx than in the thyroid precursor cells where it is maintained throughout development to adult life. *Foxe1*-null mice die shortly after birth due to a severe cleft palate phenotype. While the thyroid bud forms normally in these mice, the precursor cells are unable to migrate to the tracheal position at E9.5 and remain in the pharyngeal endoderm (6). *Foxe1*-null mice exhibit one of two thyroid phenotypes: athyreosis or ectopic thyroid (usually with a sublingual position). In contrast to mice, no thyroid ectopy has been observed in the absence of *foxe1* in zebrafish (17). Furthermore, in humans no case of thyroid ectopy due to mutations in *FOXE1* has been described to date, with the exception of polymorphism in its polyA tail (18). Based on this, it has been proposed that the differences between the two phenotypes might be due to stochastic events or to variations in the genetic backgrounds or sex-related factors of the mice (19). There is evidence, however, to support the potential role of Foxe1 in the migration of thyroid cell precursors from the anlage to their final position in the trachea. For instance, the reintroduction of *Foxe1* exclusively in precursor cells recovers the proper gland position in *Foxe1*-null mice, indicating a role for this factor as the main regulator of thyroid migration (19). The active migration of the thyroid cell precursors is still a matter of controversy, as it has been described that these cells do not undergo the classical epithelial to mesenchymal transition during morphogenesis (20). But, there is other evidence to suggest that Foxe1 directly regulates the expression of *Msx1* and *Tgfb3*, which are involved in the epithelial to mesenchymal transition and, consequently, in cell migration (21). It is plausible that both active migration by a novel mechanism and other morphogenetic events are involved in the final positioning of the thyroid gland. Thus, while further studies are needed there are strong indications to suggest that Foxe1 controls the migration of thyroid precursors to their definitive position in the anterior neck region on both sides of the trachea (6, 22). Another question that remains to be answered is whether migration is a necessary process for the terminal differentiation of thyroid gland as *Foxe1*-null mice, with a sublingual thyroid, express Tg (6).

### Paired Box 8, Pax8

*Pax8* was initially described in mouse kidney and later in thyroid, which is the only endoderm-derived organ where it is expressed. At E8.5, and coincident with the time of specification, *Pax8* is expressed concomitantly with *Nkx2-1* in the thickening primordium in the midline of the pharyngeal floor. Its expression is maintained in the thyroid, as well as in the kidney, ureter and genital tract, from all stages of embryonic development to adulthood. It is also expressed transiently in the brain during development but not at later, adult stages. *Pax8*-null mice are born alive, but show growth retardation likely due to low levels of thyroid hormones (23). The thyroid gland is devoid of follicular cells, and is therefore not functional, suggesting athyreosis. *Pax8*-null mice are also infertile and die at early stages unless administered T4, although this treatment does not recover the infertility due to a primary impairment in the reproductive system (24). The rudiment of the thyroid gland in *Pax8*-null mice contains only parafollicular cells expressing *Nkx2-1* but none of the thyroid-specific genes, and the gland fails to expand laterally and the final lobular structure is lost. This phenotype demonstrates that, in addition to being necessary for the survival of thyroid precursors, *Pax8* is essential for follicle formation and functional differentiation of thyroid cells at later stages of thyroid development. Accordingly, this gene is critical for the maintenance of the differentiated thyroid phenotype (25).

### Hematopoietically-Expressed Homeobox, Hhex

*Hhex* is a transcription factor first described in hematopoietic cells (26) and is also expressed in endoderm during early mouse embryogenesis. At E8.5, *Hhex* delimits the primordium of several organs derived from the foregut such as thyroid, liver, thymus, pancreas and lung (27). Its expression is maintained from embryos to adults. *Hhex*-null mice die in middle gestation and show a broad phenotypic spectrum. In the more severe cases, embryos fail to express *Foxe1* and *Nkx2-1* and have thyroid agenesis, suggesting that *Hhex* plays an important role in thyroid specification. However, the defect is not specific to this gland and instead is due to a general defect in the anterior endoderm (28). Moderate phenotypes have some thyroid specification with a hypoplastic and ectopic gland, and with variable *Foxe1* and *Pax8* expression along embryonic development (29). Thus, while *Hhex* is not strictly necessary for thyroid specification, it is nevertheless essential for maintaining the expression of *Pax8* and *Foxe1* and is required for correct thyroid development and for cell mobility and migration during thyroid organogenesis (29, 30). The relation among these factors is even more complex as *Hhex* is not expressed in *Nkx2-1-* and *Pax8*-null mice. Thus, Hhex is critical for thyroid morphogenesis and for maintaining the expression of other factors.

The mechanisms and molecular signals involved in the control of the four TTFs during development and adulthood will be addressed below. These signals have been experimentally assessed during development mainly using *in vitro* murine ESC and iPSC models, which have provided invaluable information on *Nkx2-1* and *Pax8*. Less information is available on *Foxe1*, and a paucity of studies has addressed the regulation of *Hhex*. In the adult context, the bulk of information has been provided using models of thyroid cells in culture.

### New Transcription Factors Identified in Thyroid Differentiation

While the aforementioned TTFs define the fate of precursors cells toward a differentiated thyroid phenotype, new regulatory factors continue to be identified acting upstream or parallel to these, to control thyroid differentiation. Although their role in thyroid differentiation has yet to be defined in detail, these new factors have been identified through analysis of the thyroid phenotype in mice deficient for the genes, or through their conserved function in the development of endoderm-derived organs. Below, we will give a brief overview on the role of these new factors in thyroid development and what is currently known about their involvement in adult thyroid differentiation.

Among the factors that contribute to thyroid differentiation, genes of the **Hox family** of transcription factors play an important role in the foregut during embryonic development (31). *Hoxa* genes are expressed in the embryonic structures from which the thyroid gland is derived. Specifically, *Hoxa3*, and its paralogs *Hoxb3* and *Hoxd3* are not expressed in the thyroid epithelial cells, but instead play a role in the migration of the ultimobranchial bodies and, indirectly, in the development of the thyroid gland (32). Accordingly, mice deficient for *Hoxa3, Hoxb3* and *Hoxd3* show evident thyroid malformations with a displaced isthmus, and with only one lobe containing a low number of follicles (33). Also, *Hoxb3* has been demonstrated to regulate the expression of *Nkx2-1* (34). Finally, *Hoxa5* is expressed in the developing thyroid at E12.5 and is maintained at E15.5, but disappears by E18.5 (35). This transcription factor plays an important role in thyroid development as adult *Hoxa5*-null mice present with hypothyroidism. Furthermore, although thyroid development is normal in the embryo, small and disorganized thyroid follicles are observed in later phases of gestation with reduced expression of thyroid transcription factors and *Tg* and *Tpo* (35, 36).

Concluding, the *Hox* genes provide positional information that contributes to the initiation of gene expression in the thyroid gland.

Other members of the **Nkx and Fox family** of transcription factors have been described in the endodermal layer of the developing pharynx. *Nkx2-6¸ Nkx2-5* and *Nkx2-3* are expressed in the endoderm layer of the primitive pharynx including the thyroid anlage. While the phenotype of *Nkx2-6 and Nkx2-3* null mice does not suggest a thyroid defect, *Nkx2-5*-null mice present with a small thyroid bud expressing *Nkx2-1*, *Foxe1* and *Pax8* at E9.5, pointing to a role for *Nkx2-5* in thyroid organogenesis. However, these mice die from other causes during embryonic development (19).

Similarly, *Foxa2* (formerly called Hepatocyte nuclear factor-3β) is expressed in the primitive endoderm and is decisive for liver organogenesis (37). It is also expressed in the invaginating foregut endoderm and in endoderm-derived structures including the developing thyroid. It has been reported that changes in *Foxa2* expression levels during the establishment of the anterior foregut endoderm determine thyroid fate. Specifically, high endodermal levels of *Foxa2* are progressively downregulated to give rise to thyroid precursors (38). Accordingly, *Foxa2* expression was found to be downregulated in the thyroid lineage at the bud stage (39), although its expression is maintained in adulthood and regulates the thyroid hormone synthesis-related gene *Tpo* (40). Beyond thyroid follicular cells, Foxa2 also plays an important role in the origin and differentiation of C-cells. In contrast to follicular cells, C-cells arise from precursors expressing *Foxa2*. Furthermore, the expression of this transcription factor is maintained in differentiated C-cells, and changes to its levels are involved in the progression of medullary thyroid cancer (2). For this reason, it can be acknowledged that Foxa2 plays an important role in the establishment of thyroid fate, both of the follicular cells and the C-cells of the endodermal precursors from which they derive.

In recent years, the transcription factor *Glis3* (Gli-similar protein 3) has emerged as important in thyroid development, as mutations in this gene have been related to congenital endocrine defects, including thyroid alterations. Specifically *GLIS3* mutations are associated with congenital hypothyroidism (41, 42).

***Glis3*** belongs to the Krüppel-like subfamily of zinc finger transcription factors (42) with variable-length transcripts generated by alternative splicing in a tissue-specific manner. The larger transcripts are highly expressed in kidney, thyroid and endocrine pancreas, whereas the smaller transcripts are expressed in kidney, heart and liver (43, 44). Beyond changes in the expression pattern, there is no evidence of functional differences between the splice variants. Similar to other transcription factors, most of the information regarding *Glis3* comes from murine models. *Glis3*-null mice die soon after birth due to neonatal diabetes, as this factor is responsible for pancreatic β-cell development (45). In addition, *Glis3*-null mice have polycystic kidney disease, which suggests a role in renal function (46).

In the thyroid gland, *Glis3* is expressed exclusively in follicular cells, and not in the parafollicular compartment, and it is required for TSH induction of thyroid hormone biosynthesis and thyroid cell proliferation in postnatal thyroid development. Although *Glis3*-null mice develop hypothyroidism, no other major complications have been found in gland development (47).

*Glis3* encodes a protein of 90 kb, which acts as an activator of gene transcription by binding to specific sites present in the regulatory regions of target genes (48). One of these target genes is *Slc5a5* (encoding the Nis symporter) and Glis3 binds to its promoter and functionally cooperates with Pax8 in a manner that does not involve the interaction between the two. Furthermore, the expression levels of other genes involved in thyroid hormone biosynthesis, such as *Slc26a4*, *Slc16a4, Tpo*, *Tg, Tshr* and *Duoxa1* and *2*, show some variability in null *Glis3*-null mice (47), as reviewed in (49).

Knockdown of *glis3* in zebrafish embryos also results in thyroid specification defects related to the reduced expression of thyroid markers such as Tg and Nis. Indeed, this model recapitulates thyroid dysgenesis and demonstrates that *Glis3* might determine endodermal cell fate by signaling downstream of the Sonic hedgehog (Shh) pathway (50). Several studies support the role of Glis3 in the Shh pathway. Glis3 degradation is prevented by its binding to suppressor of fused (Sufu), a negative regulator of the Shh pathway, and this interaction moderately inhibits its transcriptional activity (51). Both Shh and Glis3 are described to be localized to the primary cilia (46), which is suggested to play a role in thyroid follicular cell function due to its presence during development and permanence in adult life (52).

While thyroid cancer is not the focus of this review, it is interesting that the *GLIS3-PAX8* fusion is associated with a rare thyroid neoplasm termed hyalinizing trabecular tumor, which is characterized by the development of a trabecular pattern and intra-trabecular hyalinization in the thyroid (53, 54). This inter-chromosomal rearrangement leads to the overexpression of GLIS portions with an intact DNA-binding domain, yielding higher GLIS3 transcriptional activity during the evolution of the pathology. Indeed, *SLC5A5* and other genes related to thyroid hormone synthesis were found upregulated in hyalinizing trabecular tumors. Re-enforcing the link between GLIS3 and SHH, a recent study revealed that the oncogenic effects of the *GLIS3-PAX8* fusion were mediated through the SHH pathway, and inhibition of this pathway partly rescued the malignant features of trabecular tumors (55). These data open the door to the investigation of Glis3 in thyroid development and function.

**Sox9** is another transcription factor that has recently gained relevance in thyroid development, and is known to play a fundamental role in the development of numerous endoderm-derived organs. Sox9 belongs to the sex-determining region Y (SRY)-related high-mobility-group (HMG) box containing (SOX) family of transcription factors, and more specifically to the subgroup E (56).

The HMG box contains two nuclear localization signals and one nuclear export signal, which ensures a diverse subcellular distribution (57, 58). Sox9 plays a critical role in the development of the pancreas and lungs (59), and is important for maintaining a pool of undifferentiated progenitor cells in some adult tissues (60, 61).

Regarding thyroid development, the group of Nilsson has recently shed light on the function of Sox9 by showing that it is expressed at E9.5 in Nkx2-1-positive thyroid precursor cells. At E10.5, *Sox9* expression is observed only in those cells that are part of the thyroid bud, specifically in the distal population, but not in those Nkx2-1 positive cells that have been retained in the thyroid placode and have not invaginated from the anterior endoderm. Thus, Sox9 is a transcription factor with a dynamic expression during thyroid budding, which confirms early ideas that essential transcription factors are differentially regulated in thyroid progenitors transiting from the placode to the bud (29, 62). In addition, it has been described that at this stage the mesenchyme surrounding the thyroid primordium expresses high levels of Sox9, which has led to propose that it could also be regulating thyroid development in a non-cell autonomous manner (62).

Branching is a fundamental mechanism in the embryonic development of various organs, typically in exocrine and ductal organs. Through this mechanism, they acquire the appropriate size and organization. Sox9 plays a role in the regulation of the developmental branching processes of several organs such as the lungs (63) and salivary glands (64). Developmental branching of the thyroid occurs after the migration of the gland to its final position, specifically after bilobation and fusion with ultimobranchial bodies, but prior to follicular cells differentiation and Tg production (62). In this process, the cells with the highest level of *Sox9* expression are located in the distal zone of the expanding lobe, as occurs in other branching organs (63, 64). This disposition suggests that Sox9 has a role controlling the branching program of the developing thyroid gland during the stage of pre-follicular growth. This process has been recognized as reminiscent of the exocrine origin of the thyroid gland, as in the case of the endostyle of protochordates. This hypothesis is supported by the fact that thyroid follicular cells are most likely the only endocrine cells that present a clear apical-basal polarity and secrete their product to an interior space, in this case the lumen of the thyroid follicles (4). The branching process and *Sox9* itself are finely regulated by growth factors. Among them, Fgf10, generated in the surrounding mesenchyme, and its receptor Fgfr2b, have been proposed to be involved in branching and organogenesis in several organs (65, 66) including the thyroid. Unlike what occurs in other endodermal organs, such as the pancreas, Fgf10 does not control the expression of Sox9 in the thyroid progenitors. However, the dual role of Fgf10 inducing the proliferation of progenitor cells and stimulating the branching of the developing thyroid has been shown to be essential (62). An animal model with a totally inhibited expression of *Sox9* specifically in the thyroid is needed to study in-depth the role of Sox9 in the embryonic development of this gland. Currently available models, such as the generated with *Cre* under the control of the *Nkx2-1* promoter (*Nkx2-1Cre;Sox9^fl/fl^*), have failed to inhibit *Sox9* expression sufficiently to assess its function (62). In conclusion, a new and interesting role of Sox9 has been proposed in thyroid embryogenesis, which should fuel further study of the role this transcription factor not only in thyroid development but also in differentiation

In addition to the above transcription factors, other cofactors such as coactivators or corepressors also play important roles in thyroid differentiation, both in the embryo and in the adult. One of those cofactors is **Eya1** (Eyes absent 1), which has a role in the morphogenesis of organs and tissues derived from the embryonic pharynx, including the thymus, parathyroid and the thyroid. *Eya1* is expressed in the third and fourth pharyngeal region and in the ultimobranchial bodies. *Eya1*-null mice have persistent ultimobranchial bodies, hypoplasia of the thyroid lobes, no isthmus, and show a substantial reduction in the number of follicular cells and C-cells (67). *Eya1* is not expressed in the thyroid diverticulum, which suggests that defects in follicular cells are due to the lack of fusion of the ultimobranchial bodies to the thyroid lobes. This idea is supported by the finding that *Eya1*-null mice do not show alterations in early thyroid development, with normal expression of *Nkx2-1* and *Pax8*.

Interestingly the expression of *Six1*, the main transcription factor co-activated by Eya1, is severely reduced in the pharyngeal endoderm of *Eya1*-null embryos (67); however, its role in thyroid development remain to be elucidated.

Finally, another cofactor with an important role in thyroid function, both in development and adulthood, is **Taz** (transcriptional coactivator with PDZ-binding motif, encoded by the ***Wwtr1*** gene), which is mainly recognized for driving the transcriptional output of the Hippo signaling pathway. Hippo cascade activation has a key role in controlling tissue homeostasis and organ growth by regulating many cell processes such as proliferation, apoptosis, and differentiation. Nucleus-cytoplasmic shuttling of TAZ ultimately determines its transcriptional activity and is dependent on its phosphorylation state. In its non-phosphorylated form, TAZ is located in the nucleus where, as a cofactor, it cannot directly bind DNA. Instead, TAZ interacts with different transcription factors to enhance or repress their transcriptional activity. Specifically, its main interactor partners are members of the Tead family of transcription factors (68). *WWTR1* is expressed in all tissues, with the exception of the thymus and peripheral blood leukocytes (69). In rat thyroid follicular cells, Taz is found mainly in the nucleus where it has been described to interact with Pax8 and Nkx2-1 (70). During mouse thyroid development, *Wwtr1* expression is detectable from E14.5 through adult life, concomitantly with *Tg*. Taz is described to co-activate both Pax8 and Nkx2-1 in the regulation of *Tg*, although the studies leading to these conclusions were performed exclusively in a heterologous system (70). TAZ nuclear enhancement has been used to promote thyroid differentiation in activin A-treated human ESCs, as TAZ activation by the DNA-intercalating agent ethacridine triggers upregulation of *PAX8* and *NKX2-1* expression (71).

*Wwtr1*-null mice show a polycystic kidney phenotype and alveolarization defects, and although no thyroid defects have been characterized in these animals, their small size suggests a hypothyroid phenotype (68).

Similar to what is seen in mice, *taz* expression can be observed in the thyroid primordium in zebrafish larvae. Morpholino-mediated knockdown of *Wwtr1* in zebrafish results in a lower number of (smaller) thyroid follicles at later stages of development, suggesting that the co-factor is necessary for thyroid growth in this animal model. Nevertheless, its effects seemed to be unrelated to *pax8* and *nkx2-1* regulation, as its absence was found to trigger higher levels of thyroid-specific genes such as *tg, slc5a5* or *tpo* (72). While the mechanism by which Taz transcriptionally enhances Pax8 and Nkx2-1 is not fully understood, analysis using an assay for transposase accessible chromatin sequencing (ATAC-seq) showed that chromatin accessibility of TAZ, PAX8 and NKX2-1 was enriched after TAZ activation by ethacridine exposure during human ESC differentiation (73). Interestingly, TAZ was described to interact with GLIS3 in kidney cells, and their co-expression was found to promote TAZ nuclear translocation and mediate co-activation of GLIS3 transcriptional activity (46). Considering the importance of both factors for the onset of thyroid differentiation, a possible interplay between these two proteins should be investigated. Despite the promising results indicating a role for Taz as an inductor of thyroid differentiation, there has been no characterization of its effects. Nevertheless, the available data suggest that Taz could be a modulator of thyroid morphogenesis, and may control the size of the gland, as has been described for the Hippo pathway in other organs (74–76). Further research is needed to confer a specific role for this co-factor and its paralog Yap in thyroid development.

### Cross-Talk Between Transcription Factors Involved in Thyroid Development

**Figure 4:**
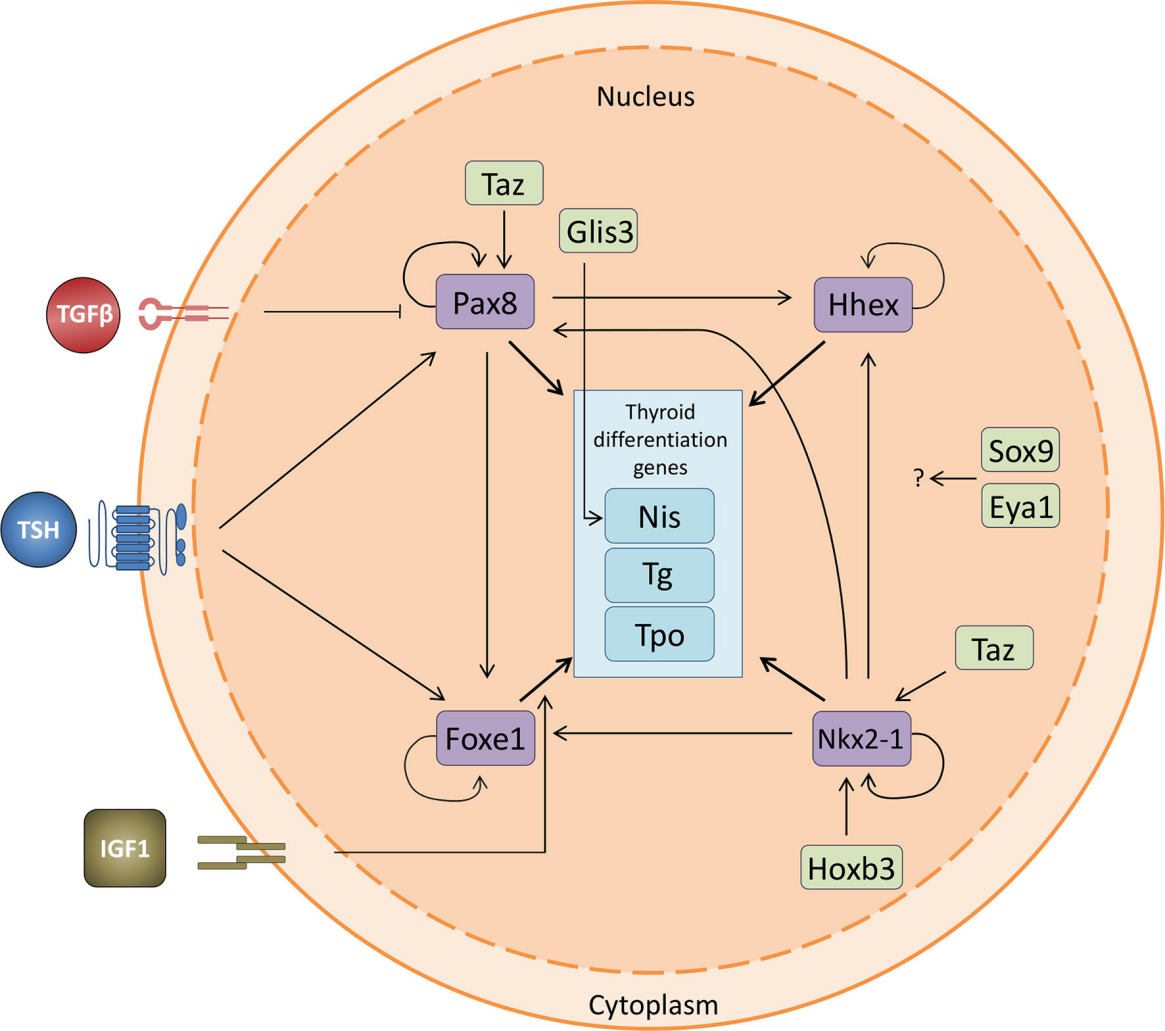
Thyroid transcription factor crosstalk. Relationship between the four thyroid transcription factors and the thyroid differentiation genes, including the interaction with the novel transcription factors involved in thyroid differentiation. *Nkx2-1* is regulated by Taz and Hoxb3 and promotes the expression of the other three TTFs. Pax8 regulates the expression of *Hhex* and *Foxe1* and is positively regulated both by TSH and Taz. It is also negatively regulated by TGFβ. *Foxe1* is positively regulated by TSH and IGF1. All four TTFs promote the expression of the thyroid differentiation genes *Nis (Slc5a5*), *Tg* and *Tpo*. The effect of Sox9 and Eya1 remains to be fully elucidated. Pointed arrows denote activation while flat arrows indicate inhibition.

Cross-communication between the four TTFs has been suggested. While it is clear that all four TTFs are expressed in thyroid precursors, it is not conclusively accepted whether all four are necessary for the commitment of the differentiation process from a precursor cell to a differentiated thyroid cell. The expression of *Nkx2-1* and *Pax8* is sufficient to drive differentiation to a thyroid lineage in murine ESC, and *Nkx2-1* can induce *Foxe1* expression (38, 77). This, together with the fact that *Pax8* regulates *Foxe1* in mice, suggests that this latter factor is downstream in the regulatory network (29). Because Foxe1 is considered as a pioneer factor with the intrinsic property to remodel chromatin (78), it likely instructs the thyroid differentiation program responsible for initiating the expression of thyroid differentiation genes. Furthermore, as we have observed an auto-regulatory mechanism for *Nkx2-1* and *Pax8* expression in thyroid cells in culture (unpublished data), a feed-forward transcriptional loop might be operating in thyroid development. Together, these data suggest the presence of a hierarchical network among the TTFs and a relationship between them (**Figure 4**). How other factors and cofactors such as Hox, Glis3, Sox9, Eya1 and Taz, and possibly others, participate in this network has not yet been studied in detail. In addition, genes acting upstream of TTFs in the regulatory network are not well known. Identifying these genes will be necessary to fully understand the early events that control thyroid differentiation in development.

## Molecules and Signaling Pathways Involved in Early Thyroid Differentiation During Embryonic Development

While the role of transcription factors in thyroid development is becoming ever more clear, the signals that regulate their expression and function in thyroid specification during embryonic development are less understood. TSH signaling, the major thyroid differentiation and proliferation stimulus for thyroid cells (79), appears not to be involved at this stage, as *Tshr* is not expressed until E15.5 in mice (80). There have been many advances in the elucidation of signals involved in early thyroid specification, budding, proliferation and folliculogenesis, but we are still far from knowing all the answers.

Because of the close proximity of the pharyngeal endoderm to the heart primordium, thyroid bud formation might be regulated by signals emerging from cardinogenic mesoderm, as has been described for other endoderm-derived organs (81). In this context, several factors have recently been identified in studies carried out in murine ESCs and iPSCs. Among them, fibroblast growth factors **(Fgfs)** and bone morphogenic proteins **(Bmps)** are inductive signals in early thyroid development. Treatment of mouse foregut explants with Fgf2 induces thyroid differentiation and, in zebrafish, Fgf1, Fgf2 and Fgf8 treatment rescues thyroid development in mutants lacking a thyroid (82). Furthermore, the use of an induction medium containing Fgf2 and Bmp4 stimulates functional thyroid differentiation in murine ESCs and iPSCs (83), whereas blocking both signals inhibits thyroid development. These results are in agreement with the finding that mice null for *Fgf2-receptor* and *Fgf10* have athyreosis or thyroid rudiment, respectively (84, 85). In addition, the Notch pathway is involved in early thyroid development, as its disruption limits the number of progenitor cells. Notch also plays a central role during zebrafish thyroid development (86). Other morphogenic signals such as Wnt/βcatenin seem not to be involved, as thyroid development is not impaired in mice null for different components of this pathway (87). The combination of BMP4, Fgf2 and Wnt3a added to a medium developed for the directed differention of ESCs resulted in maximum efficiency of Nkx2-1-positive cell specification (83). Interestingly, Nkx2-1-positive cells cultured in the presence of Fgf2 and BMP4 express thyroid differentiation markers, whereas the same cells cultured with Wnt3a and BMP4 express lung differentiation markers (88). These data suggest a role for the Wnt pathway in lung differentiation.

Shh is not involved in thyroid specification since *Shh*-null mice present with a phenotype of thyroid hypoplasia and hemiagenesis (89). Shh is expressed in the ventral endoderm except in the areas forming the thyroid bud (29, 89). These data suggest that this signaling pathway regulates thyroid development indirectly by controlling the shape of the pharynx and its vasculature. Interestingly, in addition to thyroid hypoplasia the thyroid fails to fuse with the ultimobranchial bodies in *Shh*-null mice (4).

Likewise, the transcription factor Tbx1, regulated by **Fgf8** in the developing mesoderm, stimulates the dissemination of thyroid precursors after they exit the endoderm (90, 91). In this regard, *Fgf8*-null mice show hypoplastic thyroid primordium, whereas *fgf8*-deficient zebrafish have a smaller thyroid with a lower number of follicles (82). Interestingly, the hypoplastic thyroid phenotype of *Shh*-null mice is similar to that of *Tbx1*-null embryos, indicating that the Shh gradient induced in the pharyngeal endoderm elicits distal effects that later promote proliferation of thyroid progenitors (4). It is important to note that *Shh* is expressed in the endoderm that surrounds the thyroid primordium during development, but not in the thyroid precursors themselves, and also that *Shh*-null mice – which show hypoplasia and hemiagenesis – express *Tg* (89). These data confirm that Shh signaling is involved in thyroid morphogenesis and localization, but not in the final differentiation program.

Once thyroid precursor cells have reached their final destination at the front of the trachea, the complex process of folliculogenesis begins. Despite the importance of follicle formation for thyroid differentiation, little is known about the signals involved in this process. It is currently accepted, however, that intrinsic transcription factors and extrinsic signals play an important role. Regarding transcription factors, the over-expression of *Nkx2-1* and *Pax8* in murine ESCs induces functional follicle formation in a 3D culture system (77). These data support earlier work showing that mice expressing *Nkx2-1* mutated in phosphorylation sites present with a thyroid phenotype with impaired follicular organization and a reduction in the expression levels of *Cadherin 16 (Cdh16)* and other proteins essential for follicular formation (92). Pax8 also seems to play a role in folliculogenesis as *Pax8*-null mice lack follicles (23), and the absence of Pax8 in a polarized Fischer rat thyroid cell line hinders the acquisition of apical-basal polarity and results in impaired lumen formation (93). The control of thyroid polarity by Pax8 is mediated by Cdh16 and laminins, with the latter also involved in follicle development in the mouse embryo induced by Bmp through Smad1/5 (94). In addition to Bmp, signals originating from the surrounding thyroid bud are essential for folliculogenesis (19). Among others, vascular endothelial growth factor A (Vegfa) has an important role in this process. During embryonic development, reciprocal communication through signaling molecules between endothelial cells and thyroid precursors is essential for the maturation and formation of thyroid follicles (95). Thyroid progenitors have been reported to secrete VEGFA, inducing endothelial cell recruitment and the formation of the vascular network that surrounds the thyroid follicle. Endothelial cells establish the correct follicle-shaped structure of thyrocytes due to the transmission of molecular signals through extracellular vesicles. It has been shown that, in particular, laminin-α1 contained in the extracellular vesicles that are taken up by thyrocyte progenitors plays an important role in folliculogenesis and in the formation of the angiofollicular unit (96). Nevertheless, the mechanisms involved in follicle formation need to be studied in more depth.

The role of TSH in follicle formation remains controversial. While it seems to play no role in *de novo* follicle formation, as a correctly formed thyroid gland with follicles is observed in both *Tsh-* and *Tshr*-null mice (80), it is needed for follicle formation in murine ESCs cultured in 3D overexpressing *Nkx2-1* and *Pax8* (77, 83).

## Control of Thyroid Differentiation at the Last Stages of Embryo Development and in Adult Follicular Cells

Once thyroid precursor cells have completed their migration to the trachea, and after bilobation and formation of follicles, the final process of thyroid differentiation ensues, which is maintained along adult life. Because thyroid function is under spatial and temporal control, thyroid differentiation genes are expressed following a strict developmental program: *Tg* is the first expressed at E14.5, *Tpo* and *Tshr* at E15 (12) and *Slc5a5* and *Duox2* at E15.5 (97, 98); T4 is detected at E16.5 (80). These embryonic periods in mice and the equivalent times in humans are depicted in **Figure 1**.

As explained earlier, the functional unit of the thyroid gland is the follicle, a structure formed by thyroid epithelial cells surrounding a lumen and tasked with the synthesis and secretion of T3 and T4 (**Figure 2**). As both T3 and T4 are iodinated hormones, iodide is the limiting factor for thyroid hormone synthesis. Iodide enters the thyroid cell from the bloodstream through a specific symporter located on the basolateral membrane of the follicular cell, Nis (97). From there, it is transported to the apical membrane, where it passes to the lumen by the action of Pendrin and other transporters that are still not well defined (99). In the apical membrane, iodide is oxidized by Tpo in an enzymatic reaction that requires H_2_O_2_ generated by Duox2. In the lumen, Tg synthesized by the follicular cells is iodinated and stored in the colloid. Iodinated Tg is the protein matrix for the formation of mono- and diiodotyrosine (MIT and DIT) and their coupling to generate T3 and T4. Depending on the need for thyroid hormones, iodinated Tg is endocytosed in the form of colloid drops, fused with lysosomes and proteolyzed to release MIT, DIT, T3 and T4. Iodide is recycled from MIT and DIT by Dehal1, encoded by *Iyd1*, and T3 and T4 are transported to the bloodstream by Mct8 located on the basal membrane.

### Regulation of Thyroid Differentiation Genes

All processes involved in the synthesis and secretion of thyroid hormones require the exquisite regulation of the differentiation genes. The major regulator of thyroid function is the pituitary hormone TSH, and its role is co-ordinately controlled by other factors, among which are IGF1 and TGFβ.

#### Thyroid Stimulating Hormone (TSH)

TSH, a glycoprotein hormone synthesized and secreted by the anterior pituitary, is the major stimulus that controls proliferation and differentiation at late developmental stages and in the adult thyroid gland (79, 100). TSH controls thyroid hormone production and serum concentration through a negative feedback loop by binding to its receptor (TSHR) to signal through a number of pathways, targeting transduction and transcription effectors. TSHR belongs to the G protein-coupled receptor (GPCR) family and is anchored to the basal membrane of follicular thyroid cells. It is composed of two subunits, α and β, which are non-covalently linked (101, 102). Stimulation of TSHR induces dissociation of heterotrimeric G proteins leading to Gα and Gβγ activation. There are many subtypes of α and βγ subunits and their combinations activate different downstream pathways. In thyroid cells, Gα-induced subunits are mainly Gαs, whose activation “stimulates” adenylate cyclase to elevate cAMP production (103, 104). As a second messenger, cAMP has cell type-specific effects and acts principally through cAMP-dependent protein kinase A (PKA), which is necessary but not sufficient to reproduce the full mitogenic effect of cAMP in some thyroid cells (105). The catalytic subunit of PKA phosphorylates its target proteins including the transcription factor CREB, which is its classic nuclear target (106, 107). Phosphorylation of CREB recruits coactivators such as CBP (CREB-binding protein) and p300, enhancing the activity of cAMP response elements (CRE)-containing gene promoters. The transcriptional activity of CREB and, consequently, the activation of the TSH signaling pathway, are downregulated by its dephosphorylation, mediated by the action of PP1 and PP2A phosphatases (108). The cAMP-mediated TSH signaling pathway and the resulting activation of CREB is essential for the maintenance of the differentiated state of the follicular cell and therefore for the correct function of the thyroid (109). Interestingly, it has been described that mice with specific suppression of CREB lack thyroid follicular structures and show no expression of thyroid-specific proteins (110). Although the phosphorylation of CREB by cAMP/PKA is the primary event elicited by TSH, other transcription factors such as CREM can mediate the TSH/cAMP response in the regulation of *Slc5a5* expression (111). As the TTFs are phosphoproteins (112–114), many studies have been performed to dissect the role of post-translational modification in the regulation of thyroid-specific genes. Thus, it has been reported that TSH, through cAMP, can induce the phosphorylation of TFFs and therefore their transcriptional activity. Nkx2.1 phosphorylation is important for its binding and activity to regulate TSHR transcription (115). However, other work reported that Nkx2.1 phosphorylation is not affected by cAMP treatment in dog thyrocytes (116). In addition, Nkx2.1 phosphorylation at serine 337, in response to an increase in cAMP levels, is not necessary for its activation of the *Tg* promoter (117). From the last two reports, it can be concluded that other transcription factor(s) or co-factor(s) are involved in the control elicited by cAMP/PKA on the expression of thyroid-specific genes. Pax8 is a nuclear phosphoprotein and it has been reported that its mRNA and protein levels are cAMP dependent; however, neither its phosphorylation nor its translocation to the nucleus are regulated by cAMP in dog thyrocytes (118). Furthermore, it has been shown that cAMP stimulation increases *Pax8*-specific transcription in rat thyroid cells, a mechanism that could be due to a direct target of PKA activating Pax8 (113). The phosphorylation of Foxe1 and Hhex in response to TSH/cAMP has been studied to a lesser extent and no information is yet available.

Overall, we can conclude that there is no clear demonstration that TSH/cAMP/PKA directly phosphorylates TTFs. Instead, the most plausible idea is that this signaling pathway could phosphorylate other factors co-operating with the TTFs.

#### Thyroid Differentiation Genes Regulated by TSH

As the main regulator of thyroid differentiation, TSH controls, at the transcriptional level, all of the genes that define the thyroid-differentiated phenotype: *Tg, Tpo, Slc5a5*, *Tshr* and *Slc26a4* (7, 8, 119). As the bibliography on the transcriptional regulation of these genes is extensive, here we will discuss only the role of CREB and the TTFs in the TSH-mediated regulation of *Tg*, *Tpo* and *Slc5a5*, which has been studied more extensively in rat thyroid cells. Because TSH/cAMP regulates all three genes, they should contain CRE sites in their upstream regulatory region. Yet, no consensus CRE motif has been identified, except in *Tg* and in the *Nis* promoter/enhancer where a CRE-like motif has been described (9, 120–122). This would suggest that specific transcription factors modulate the TSH-regulated transcription of these genes.

In this regard, TSH induces the expression of the TTFs Pax8 (123) and Foxe1 (122, 124). Less clear is the role of TSH on Nkx2-1, as no effect (116) or a downregulation (125) has been reported. TSH not only regulates TTF gene expression but also the binding to their consensus sites within target promoters (9, 16, 80, 126–130). The mechanism of this regulation should be dependent on TTF phosphorylation; however as explained earlier it is currently not clear whether the phosphorylation state has a role to play. By contrast, what is accepted is the dependency of the redox state of these factors (131). For instance, the ability of Pax8 to bind to its consensus site in the *Slc5a5* promoter depends on the reduction of two highly conserved cysteine ​​residues that are found in all the proteins of the Pax family (132). Ape1 endonuclease (also known as Ref-1), whose expression is TSH-dependent, together with thioredoxin (Txn) and thioredoxin reductases (TxnRd1, 2 and 3), are the principal enzymes responsible for the reduction of Pax8 (133) and, consequently, the activation of the binding capacity of Pax8 to DNA through a selenium-dependent mechanism (134).

It is important to note that there are differences in the control of TSH action in the different mammalian species studied (135). With the exception of dog thyrocytes in culture, TSH also activates the phospholipase C signaling pathway, which leads to the organification of iodide. Furthermore, in some thyroid cell systems, TSH also activates MAPK and PI3K pathways; specifically, TSH has been shown to activate ERK (136), mTOR1 (137) and PI3K by signaling the RAS GPTase (138, 139). Furthermore, TSH induces the release of Gβγ dimers upon binding to its receptor, resulting in the activation of the PI3K/Akt pathway. Activation of this pathway homeostatically counteracts the effect induced by increased cAMP levels, as in the case of Nis (140).

This combined evidence indicates that the effects of TSH on the proliferation and differentiation of the thyroid follicle cell occurs *via* a complex network of cross-communication between different signaling pathways and their associated effectors, which is different among species. This is important to consider when evaluating the pathogenic effect that alterations of these signaling pathways has on each species, and is also important when extrapolating results between species.

#### Insulin-Like Growth Factor 1 (IGF1)

IGF1 is a growth factor similar in its molecular structure to insulin, and plays an important role in body growth by acting on almost every cell type. Many mechanisms are regulated by IGF1, including DNA synthesis, proliferation and differentiation (141–143). IGF1 binds to its transmembrane receptor tyrosine kinase (IGF1R) on the cell surface, which promotes its dimerization and autophosphorylation resulting in insulin receptor substrate-1 (IRS-1) and IRS-2 phosphorylation and PI3K recruitment to the cell membrane, in turn activating the PI3K signaling pathway (144–147). Active PI3K then catalyzes the formation of phosphatidyl-inositol triphosphate (PIP3), which leads to the recruitment of AKT and its activator PDK1 to the cell membrane. Two downstream components of this pathway – p27^KIP1^ and FOXO1– whose nuclear exclusion is a hallmark of the mechanism of action of IGF1, are involved in the function of IGF1 in thyroid cell proliferation and differentiation (148, 149).

Binding of IGF1 to its receptor also results in the phosphorylation of Src homology collagen (Shc), which leads to the activation of RAS and the subsequent activation not only of PI3K, but also of MAPK through the sequential activation of MEK and ERK. In turn, ERK activates several targets along the different compartments of the cell especially in the nucleus where it phosphorylates effectors related mainly to proliferation such as c-Fos, c-Jun, c-Myc or ELK1 (150).

#### Thyroid Differentiation Genes Regulated by IGF1

While little is known about the role of IGF1 in thyroid development, it is a recognized modulator of the TSH response in adult thyroid cell differentiation. IGF1 increases TG and TPO expression levels and has an additive effect with TSH (151, 152). This effect occurs at the transcriptional level, as IGF1 stimulates *Tg* and *Tpo* promoter activity *via* increased binding of Foxe1 to both promoters (126, 127). Accordingly, Foxe1 is at the center of the transcriptional control of both thyroid differentiation genes since, as explained above, it also mediates TSH regulation (122, 124).

However, IGF1 does not always play a positive role in the regulation of thyroid differentiation, as is the case for *Slc5a5* in which IGF1 has been described to repress the TSH-induced expression involving the PI3K pathway (153). All of the aforementioned mechanisms of action of IGF1 make this growth factor an important component necessary for regulating follicular cell differentiation and modulating TSH action.

#### Transforming Growth Factor β (TGFβ)

TGFβ1 is a homodimeric protein of the TGFβ subfamily of cytokines, secreted by many cells types, that regulates cell proliferation, survival, adhesion, migration, differentiation and specification of developmental fate. TGFβ1 binds and brings together two different types of serine/threonine kinase receptors: TGFβR I and II. This allows TGFβRII to activate TGFβRI (also known as ALK5) by phosphorylation of specific cytoplasmic domains (154). In canonical TGFβ signaling, active TGFβRI phosphorylates the receptor-regulated SMADs (R-SMADs) 2 and 3 at two C-terminal serine residues. SMAD-4, the unique co-SMAD, binds to SMAD2/3 upon their phosphorylation, forming a heterotrimeric transcriptional complex that translocates to the nucleus (155). In the nucleus, SMADs bind to different DNA-regulatory regions by interacting with other transcription factors, transcriptional co-repressors, and co-activators, to inhibit or activate gene transcription. Furthermore, this signaling network is subject to negative feedback provided by the inhibitory SMADs (I-SMADs) such as SMAD-7, which suppresses R-SMAD and TGFβR activity by (156).

#### Thyroid Differentiation Genes Regulated by TGFβ

TGFβ1 is synthesized and secreted by thyroid follicular cells, where it blocks the effects of TSH/cAMP on cell proliferation (157–159), cell shape (160, 161) and differentiation (162), thus contributing to thyroid gland homeostasis. TGFβ diminishes the mRNA levels of the differentiation genes *Tg, Tpo* and *Slc5a5* by a mechanism transcriptionally regulated by Pax8 downregulation (163–165). Smad2/Smad4 are the main players of this inhibition in the case of *Tg*, whereas Smad3-Pax8 interaction drives *Slc5a5* downregulation. Additionally, TGFβ1 also affects Na^+^/K^+^ ATPase activity (166) and Tshr levels (167), both crucial for the iodide uptake needed for thyroid hormone synthesis. Overall, these data establish TGFβ1 as a negative regulator of differentiation maintenance in the adult thyroid.

TGFβ1 also participates in the de-differentiation processes that occur in papillary thyroid carcinomas. The BRAF-V600E oncogenic driver triggers TGFβ1 secretion, which represses *SLC5A5* transcription by a mechanism that involves NOX4-derived reactive oxygen species generation by Smad signaling (168, 169). This contributes to impairment in iodide uptake, which leads to radioactive iodide refractory disease and a worse therapy outcome.

Activin A, another member of the TGFβ subfamily, is also expressed in thyroid follicular cells. By engagement with activin type IB and type II receptors, activin A is reported to induce SMAD2-3/4 complex translocation to the nucleus, thus interfering with the TSH-mediated cAMP response. The effects of activin A have been described to be milder than those of TGFβ1 (167).

## Conclusions Remarks and Future Perspectives

The mechanisms involved in the development of the thyroid gland are dynamic and complex, and are increasingly better understood, particularly in terms of specific TTFs and other factors upstream or interacting with them to ultimately modulate their function. Knowledge of signaling factors has contributed to a better understanding of the development of this gland and, consequently, the formation of the thyroid hormones, and both animal models and stem cells have been critical for this advance. In this review, we have attempted to summarize the most current knowledge. Although much progress has been made, in our opinion there has been a paucity of studies in recent years. It is possible that this is because many research groups focus more on studies of thyroid pathologies. However, without basic studies, it is challenging to obtain information on these pathologies. Knowledge of thyroid development in animal models has contributed to a richer understanding of physiologic and pathologic processes, including congenital hypothyroidism and cancer. Accordingly, the future should be aimed at developing new animal and ESC models, and even 3D bioprinting of the thyroid gland. Indeed, this has already been developed (170, 171) and has the potential to greatly aid our understanding of all the associated processes, including the vascularization of the gland. Furthermore next generation sequencing will be instrumental in identifying new genes, and epigenetic analysis will help to create new concepts.

## Author Contributions

AL-M, CC-L, and CF-M, wrote the different sections of the manuscript. PS prepared and edited the final manuscript. All authors contributed to the article and approved the submitted version.

## Funding 

This work was supported by grants PID2019-105303RB-I00 from Ministerio de Ciencia e Innovación (MICIN), Spain, S2017/BMD-3724 from Comunidad de Madrid, and GCB14142311CRES from Fundación Española Contra el Cáncer (AECC).

## Conflict of Interest

The authors declare that the research was conducted in the absence of any commercial or financial relationships that could be constructed as a potential conflict of interest.
